# Bronchoalveolar-Lavage-Derived Fibroblast Cell Lines Provide Tools for Investigating Various Interstitial Lung Diseases

**DOI:** 10.3390/cells11142226

**Published:** 2022-07-18

**Authors:** Siri Lehtonen, Riitta Kaarteenaho

**Affiliations:** 1 PEDEGO Research Unit, University of Oulu, POB 8000, FIN-90014 Oulu, Finland; siri.lehtonen@oulu.fi; 2 Department of Obstetrics and Gynecology, Medical Research Center Oulu, Oulu University Hospital, POB 10, FIN-90029 Oulu, Finland; 3 Research Unit of Internal Medicine, University of Oulu, POB 8000, FIN-90014 Oulu, Finland; 4 Center of Internal Medicine and Respiratory Medicine, Medical Research Center Oulu, Oulu University Hospital, POB 10, FIN-90029 Oulu, Finland

**Keywords:** idiopathic pulmonary fibrosis, BAL-derived cell culture, fibroblast, myofibroblast

## Abstract

Bronchoalveolar lavage (BAL) is an important diagnostic and research tool for the investigation of various lung diseases. In addition to inflammatory and epithelial cells, BAL fluid may contain a small number of stromal cells, such as fibroblasts. During the past 30 years, a number of research groups have cultured BAL-derived fibroblasts for several passages in vitro. In addition to fibroblasts, these cultures have been reported to contain fibrocytes, myofibroblasts, and stem cells. We aim to present a summary of studies that have cultured stromal cells from BAL fluid.

## 1. Introduction

For several decades, bronchoalveolar lavage (BAL) fluid has been used for research and diagnostic investigations of various types of lung disorders including interstitial lung diseases (ILD) [1]. When performing BAL, sterile saline is installed through a bronchoscope and recovered by suctioning. An international guideline gave recommendations for the performance and processing of BAL and the interpretation of BAL cellular findings in ILD [2]. The recent international guidelines on the diagnosis of various ILDs, such as idiopathic pulmonary fibrosis (IPF), hypersensitivity pneumonitis (HP), and sarcoidosis, suggested the use of BAL in certain subgroups of suspected subjects [3,4,5].

We read with great interest the recent article of Bergantini and co-workers in Cells entitled “Bronchoalveolar-lavage-derived fibroblast cell line (B-LSDM7) as a new protocol for investigating the mechanisms of idiopathic pulmonary fibrosis” [6]. The authors published the protocol for culturing a fibroblast cell line from the bronchoalveolar lavage (BAL) fluid of a patient with IPF. The cell line was characterized phenotypically, morphologically, and functionally, and compared with the commercial fibroblast cell line from a patient with IPF. In the Discussion, Bergantini and co-authors informed that few authors have attempted the isolation of fibroblasts from BAL fluid, referring to previous studies by Quesnel et al. [7] and Larson-Casey et al. [8]. We aim to provide a commentary on the study of Bergantini and co-authors [1] and present a summary of the previous studies on BAL-derived fibroblast cultures.

## 2. History of BAL-Derived Fibroblast Cultures

The history of culturing fibroblastic cells from BAL fluid dates back more than 30 years. In 1991, Elisabeth Fireman and co-authors published a successful BAL-derived fibroblast cell culture protocol, and they managed to culture the cell line for several passages [9]. Next, they derived proliferating fibroblast cell lines from 18 BAL samples that included patients with sarcoidosis, diffuse interstitial fibrosis, HP, and controls [10]. Ten years after the first successful cell culture, Fireman et al. cultured fibroblasts and myofibroblasts from the BAL fluid samples of eight patients with IPF and seven patients with sarcoidosis [11]. In all three studies mentioned above, they performed BAL for research purposes, and in the last study, they reported using a total of 150–200 mL saline with 58% recovery. A few years later, Larsen et al. investigated 12 patients with mild asthma and 17 controls, obtaining a positive cell culture from 5 patients with asthma, while 7 cases with asthma and all the controls remained negative [12]. Subsequently, using the same method, Larsen and co-authors published another study in which they investigated ten patients with systemic-sclerosis-associated interstitial lung disease (SSc-ILD) and succeeded in five cases, while five cases remained negative [13]. In their second study they also used cell lines derived from five patients with mild asthma obtained from their first study. Another study of the same study group investigated BAL fluid from nine patients with mild asthma to characterize both cultured fibroblasts and fibrocytes [14]. The studies of Larson and Nihlberg used a total of 140-mL BAL fluid samples, although the size of the aliquots was not reported. The BAL procedures were performed for research purposes in the studies of Larsen, Nihlberg, and Fireman.

Lama and co-authors attempted to culture fibroblastic cell lines from 172 BAL fluid samples from 76 patients undergoing lung transplantation—when the BAL procedure was performed for diagnostic purposes [15]. BAL cell culturing was successful in 106 cases and negative in 66 cases. They used 10–50 mL of BAL fluid for the cultures. Quesnel et al. aimed to perform cell culturing from diagnostic BAL procedures from 68 ventilated patients (acute lung injury (ALI) *n* = 17, acute respiratory distress syndrome (ARDS) *n* = 31, and other ventilated patients *n* = 20) [7], and their cell cultures were positive in 12 cases and negative in 56 cases. In a later study by the same study group, 26 patients with IPF, 9 patients with SSc-ILD, and 11 controls were investigated for cell cultures from diagnostic BAL procedures [16]. In this study, as in the study by Sato and co-workers, the focus was on fibrocytes derived from BAL [17]. In their recent study, Codullo et al. investigated fibroblastic cell lines isolated and cultured from the BAL fluid samples of four patients with SSc-ILD [18]. We have previously cultured fibroblast cell lines from 98 diagnostic BAL fluid samples from patients with various types of lung diseases, including 71 patients with different types of ILDs [19,20].

## 3. Cell Culture Protocols

Half of the abovementioned studies performed BAL for research purposes and the other half utilized a fraction of a diagnostic sample (Table 1). Whether diagnostic or not, there seems to be variation in the success rate of the cell culture as it varies from 17% to 100%. Interestingly, the studies with the most samples, namely those of Lama and co-workers and ours [15,19,20], ended up having the same success rate of 62%. Several studies reported a poor success rate with their control samples, which is very much expected since the more intact the lung lining is, the fewer stromal cells will be flushed away during BAL. We found that the success rate of the cell culture was associated with the disease of the donor, with IPF being one with a high success rate [20].

The cell culture protocols for fibroblasts from BAL samples seem to be very similar in all the studies, with some modifications. Most researchers have used Dulbecco’s modified Eagle medium (DMEM) as a cell culture medium supplemented with 10% heat-inactivated fetal bovine serum (FBS) and antibiotics. However, Fireman and Bergantini also used Roswell Park Memorial Institute medium 1640 (RPMI-1640) during the initial phase of the culture [5,9,10,11]. Quesnel and Borie report using RPMI-1640 even further [7,16] and we have used α-modified Minimum Essential Medium (α-MEM) in our studies [19,20]. All studies have used heat-inactivated fetal calf serum, but the concentration used varies from the generally used 10–20%, such as in Fireman’s studies, and 13%, as was used in our studies (Table 1). Only Bergantini et al. have reported the use of fibroblast growth basal medium (FGBM) and a combination of human fibroblast growth factors and FBS during primary culturing, but they did use RPMI-1640 supplemented with 10% FBS for the initial attachment period. After passaging, they tested both FGBM and RPMI-1640 for different patches.

**Figure 1:**
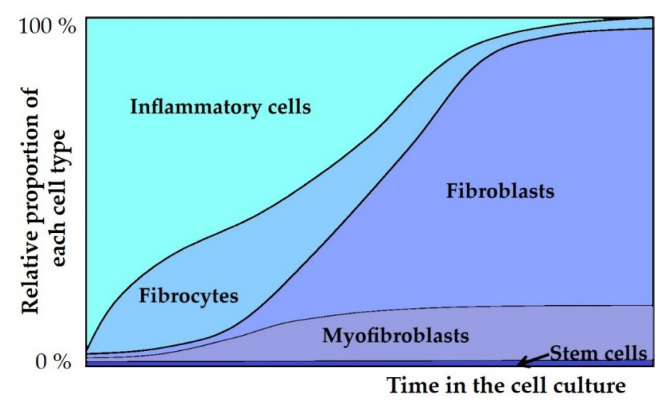
Schematic illustration of the relative proportions of various cell types during successfully-derived cell culturing. At the beginning of the culture, there are mainly inflammatory cells, but their proportion decreases rapidly. Fibrocytes are abundant during early phases and myofibroblasts become more abundant as the number of fibrocytes decreases. Fibroblasts proliferate actively and eventually overgrow other cell types. A small amount of stem cells can be seen throughout the culture.

Interestingly, Nihlberg, Borie, and Sato have used very similar cell culture methods for studying fibrocytes [14,16,17]. One difference in the study protocols was the length of the primary culture, which varied from 1 to 6 weeks and may reflect the different characterization results of different groups. In fact, Quesnel et al. found that at the beginning of the culture period, there were less than 1% of fibrocytes while fibroblasts appeared after the first week—and at 4 weeks they occasionally observed myofibroblast-type cells [7]. Nihlberg et al. reported that in their culture, about 18% of fibroblast-type cells expressed fibrocyte markers [14]. In several studies reporting fibroblast cell lines, the primary cell culture period before passaging was 3 weeks (Table 1). By choosing an appropriate time point, BAL-derived cell cultures can be used for studying various cell types as the cell profiles change over time, as illustrated in Figure 1.

## 4. Characterization of the BAL-Derived Cultured Cells

The main focus of the previously published studies has been on the characterization of BAL fibroblasts, myofibroblasts, mesenchymal stem/stromal cells, and fibrocytes, as well as on the investigation of cell function. Fireman and co-authors evaluated cytoskeleton proteins by enzyme-linked immunosorbent assay (ELISA) and immunofluorescent methods, revealing that α smooth muscle actin (α-SMA) measured by ELISA was higher in IPF than in sarcoidosis, and that only IPF revealed myofibroblast phenotypes, showing α-SMA immunofluorescence labeling and filaments with associated dense bodies with rough endoplasmic reticulum through electron microscopy [11]. Moreover, they showed that cells in IPF contracted more than those from sarcoidosis by the gel contraction method [11]. Larsen and others found that elongated fibroblasts cultured from BAL fluid were stained for the fibroblast marker prolyl 4-hydroxylase and the myofibroblast marker α-SMA [12]. Furthermore, they observed that the fibroblasts from BAL fluid migrated double the distance and produced five-fold amounts of proteoglycans compared to fibroblasts cultured from the bronchial biopsies of the same patients [12]. The protein expression pattern of fibroblasts cultured from BAL fluid was also different compared to the fibroblasts cultured from biopsies [12]. In another study of Larsen and co-authors, fibroblasts cultured from the BAL fluid of SSc-ILD patients migrated more than fibroblasts cultured from bronchial biopsies, which is similar to the results of their previous study [13]. Moreover, they found that the production of an alternatively spliced form of cellular fibronectin, ED-A fibronectin, was higher in cells cultured from BAL than in those from biopsies, and that the BAL-cultured fibroblasts expressed α-SMA and produced transforming growth factor beta (TGF-β) [13]. Nihlberg and co-authors revealed that fibroblasts from BAL fluid expressed the fibrocyte markers CD34, CD45RO, and α-SMA [14].

Lama and co-authors performed an immunophenotyping for surface antigens by flow cytometry to characterize the cell population cultured from BAL, showing that the cells strongly expressed CD73, CD90, and CD105, which are the biomarkers described in bone marrow-derived mesenchymal stem cells [15]. Moreover, they revealed that BAL-cultured cells were able to differentiate into multiple connective tissue cell lineages, such as osteocytic, adipocytic, and chondrocytic [15]. Quesnel and co-authors reported in their first study that a limited population of fibrocytes was detectable during the initial phase of BAL culturing, alveolar fibroblasts can be cultured from BAL in 25% of ALI/ARDS patients, the migration of alveolar fibroblasts of the study group was three-fold to that of the control group, and collagen I production was elevated in alveolar fibroblasts and correlated with TGF-β production [7]. In their later study, fibrocytes were detected in BAL fluid in about half of the patients with IPF and SSc-ILD [16].

We obtained 61 cell lines from BAL fluid samples, including patients with IPF, non-specific interstitial pneumonia (NSIP), connective tissue disease associated interstitial lung disease (CTD-ILD), asbestosis, pulmonary sarcoidosis, HP, drug reaction, respiratory bronchiolitis interstitial lung disease (RBILD), and desquamative interstitial lung disease (DIP) [20]. The cells were characterized by immunocytochemistry, electron microscopy, flow cytometry, and differentiation tests [19]. Our results showed that the cultured cell lines contained both fibroblasts and myofibroblasts. We visualized the cell lines cultured from 51 BAL samples by transmission electron microscopy (TEM) and immunoelectron microscopy (IEM) to achieve the ultrastructural localization of alpha-smooth muscle actin (α-SMA) and fibronectin in myofibroblasts [19]. In addition, the levels of α-SMA and fibronectin protein and mRNA were measured by Western blot analysis and quantitative reverse transcriptase polymerase chain reaction, and the invasive capacities of the cells were evaluated [19]. Furthermore, we have investigated the effect of antifibrotic drugs, namely pirfenidone and nintedanib, on the fibroblastic cell lines cultured from the BAL fluid (*n* = 4) and lung tissue (*n* = 3) samples of patients with IPF, showing that both drugs reduced the in vitro proliferation, the amount of α-SMA, and the myofibroblastic appearance of myofibroblasts [21].

We observed in our studies that the BAL-cultured cells were positive for vimentin and fibronectin, thus revealing typical biomarkers for fibroblasts. Approximately 15% of the cells were positive for α-SMA. The cells expressed surface antigens that are typical for mesenchymal stromal cells, and some cells possessed the potential to differentiate into osteoblastic and/or adipocytic lineage cells [20]. BAL fluid-cultured cell lines from 51 patients with various types of ILDs composed heterogeneous cell populations of fibroblasts and myofibroblasts. The structure of the fibronexus and the amounts of intracellular actin, extracellular fibronectin, and cell junctions of myofibroblasts varied in different diseases from electron microscopy and immunoelectron microscopy examination [19]. The invasive capacity of the cells obtained from patients with IPF was higher than that from patients with other types of ILDs, and the cells expressing more actin filaments had a higher invasive capacity.

## 5. Utilization of the BAL-Derived Cell Lines

As seen from the success rates shown in Table 1, BAL-derived fibroblast cell lines are not easy to obtain. However, despite the practical challenges, they provide several possibilities for future studies. Patient-derived cells lines cultured in vitro for a few passages represent a more natural state of stromal cells compared to commercially available immortalized cell lines that have undergone major manipulations. Even though commercial cell lines are an important tool for in vitro studies, this tool should be supplemented with experiments utilizing primary cells or cell lines derived from them. The advantage of using BAL- versus tissue-derived fibroblasts is the fact that, in this way, it is possible to obtain samples from a larger population of patients with ILDs since more patients with ILD undergo diagnostic bronchoscopy and BAL operation than surgical lung tissue or transbronchial lung cryobiopsy procedures. Obviously when using tissue samples, the success rate of the cell culture is much higher than in BAL-derived cultures. Some studies have performed BAL for research purposes, but surprisingly this does not have an effect on the success rate, as seen in Table 1. However, BAL performed for research purposes can provide an even larger donor cohort, thus allowing for follow-up studies, for example.

BAL–fibroblast cultures could be used for the investigation of the response to antifibrotic drugs, disease course, and prognosis, including the progressive nature of ILD—bearing in mind that these cells do not completely mimic the in vivo situation. A recent study of Liu, X. et al. [22] investigated pulmonary mesenchymal cells isolated from lung tissues from the developing, adult, and fibrotic lungs of mice and humans by single-cell RNA sequencing. They discovered several fibroblast subtypes in both the murine and human lung, a description which is valuable for the research community. According to several studies [10,12,14,16,20,21], fibroblast cell lines cultured from BAL seem to remain heterogeneous, but there is currently no data showing how well this heterogeneity resembles that seen in cell lines cultured from lung tissue or in lung tissue in vivo. Even though prolonged in vitro culturing affects the properties of the cells, some properties are maintained as some studies have found an association between cell characteristics and patient demographic data [7,13,14,17,20,21]. Thus, it could also be assumed that certain drugs used by donors could affect the properties of the cells. For example, drugs used for IPF treatment, namely pirfenidone and nintedanib, could have an effect on stromal cell properties. However, most BAL cells have been collected from patients at the diagnostic stage and, furthermore, most of the studies described in Table 1 have been performed before these antifibrotic drugs were available, which is why future studies are warranted.

A study of Basset et al. demonstrated that various types of ILD are associated with intra-alveolar fibrosis [23]. It can be pondered whether BAL-derived fibroblasts represent cells from intra-alveolar fibrosis rather than from the interstitial area and that BAL–fibroblast-cultured cells may reveal, at least partially, different properties than those cultured from lung tissues, a phenomenon which can be seen either as a disadvantage or advantage depending on the perspective of researchers.

## 6. Conclusions

All in all, it can be concluded that fibroblast cell cultures from BAL fluid samples of patients with various lung diseases have been studied for more than 30 years. BAL procedures have been carried out either for research or for diagnostic purposes. The number of patients in each study have varied markedly—from one to more than 100 patients—and the patients have suffered from various types of lung disorders. Furthermore, the cultured fibroblastic cells have been investigated by multiple methods. It is delightful that Bergantini and co-authors have re-introduced the BAL fluid culture method as it may offer several possibilities for the research of lung diseases. Several interstitial lung diseases, such as IPF, still lack a cure and new diagnostic tools would be beneficial. Therefore, the utilization of diagnostic BAL samples to obtain disease-specific stromal cell lines for research provides an elegant tool, as suggested by Bergantini and co-workers.

## Figures and Tables

**Table 1:** Studies with cultured stromal cells derived from BAL.

Study	Patient Diagnosis (*n*)	Purpose of BAL	Cell Culture Medium	Length of Primary Culture Period	Aim of the Cell Culture
		Volume of BALF for Cell Culture	Concentration of FBS	Passages	Success of the Cell Culture (%)
Fireman [9]	Sarcoidosis (5)	Research	RPMI-1640, after passaging DMEM	5–6 weeks	Fibroblasts
		*	10% FBS	*p*. 4–12	(20% *)
Fireman [10]	6 Sarcoidosis (6), Diffuse interstitial fibrosis (3), HP (3), Controls (6)	Research	RPMI-1640, after passaging DMEM	5–6 weeks	Fibroblasts
		*	10% FBS	*p*. 4–7	(100% *)
Fireman [11]	IPF (8), Sarcoidosis (7)	Research	RPMI-1640, after passaging DMEM	3–4 weeks	Fibroblasts and myofibroblasts
		87–116 mL *	10%, after passaging 20% FBS	*p*. 2–4	(100% *)
Larsen [12]	Mild asthma (12), Controls (17)	Research	DMEM	5–6 days	Fibroblasts
		*	10% FBS	*p*. 5–7	17%
Larsen [13]	SSc-ILD (10)	Research	DMEM	5–6 days	Myofibroblasts
		*	10 % FBS	*p*. 5–7	50%
Nihlberg [14]	Mild asthma (9)	Research	DMEM	20–30 days	Fibroblasts and fibrocytes
		*	10% FBS	*p*. 4–7	56%
Lama [15]	172 BAL from 76 lung transplant recipients: Emphysema (39), IPF (16), cystic fibrosis (10), other (11), controls (15)	Diagnostic	DMEM	3 weeks	Mesenchymal stem cells
		10–50 mL	10% FBS	*p*. 2–6	62%
Quesnel [7]	Acute lung injury (17), Acute respiratory distress syndrome (31), Other ventilated patients (20)	Diagnostic	RPMI-1640	4 weeks	Fibroblasts
		*	10% FBS	*p*. 0–3	18%
Karvonen [19] Lehtonen [20]	Sarcoidosis (17), IPF (14), NSIP (10), CTD-ILD (9), Asbestosis (8), Other (28), Controls (12)	Diagnostic	αMEM	3 weeks	Fibroblasts and myofibroblasts
		15 mL	13% FBS	*p*. 1–5	62%
Borie [16]	IPF (26), SSc-ILD (9), Controls (11)	Diagnostic	RPMI-1640	At least 4 weeks	Fibrocytes
		*	10% FBS	*p*. 0–3	37%
Codullo [18]	SSc-ILD (4)	Research	DMEM	1–3 weeks	Fibroblasts
		*	10% FBS	*p*. 2–6	*
Sato [17]	10 IPF, 13 other UIP/fNSIP, 15 sarcoidosis, 21 other	Diagnostic	DMEM	7 days	Fibrocytes
		10 mL	20% FBS	*p*. 0	*
Bergantini [6]	IPF (1)	Diagnostic	RPMI-1640, after 1st day FGBM, after passaging variable	3 weeks	Fibroblasts
		60 mL	10% FBS, after 1st day *	*	(100% *)

* Not described comprehensively. (BAL = bronchoalveolar lavage, BALF = bronchoalveolar lavage fluid, CTD-ILD = connective tissue disease associated interstitial lung disease, RPMI-1640 = Roswell Park Memorial Institute Medium 1640, DMEM = Dulbecco’s Modified Eagle Medium, FBS = fetal bovine serum, HP = hypersensitivity pneumonitis, IPF = idiopathic pulmonary fibrosis, SSc-ILD = systemic sclerosis associated interstitial lung disease, ILD = interstitial lung disease, NSIP = Non-specific interstitial pneumonia, αMEM = Minimum Essential Medium Eagle alpha modification, FGBM = fibroblast growth basal medium, fNSIP = fibrotic nonspecific interstitial lung disease).
